# Alkaline Phosphatase Immobilization on New Chitosan Membranes with Mg^2+^ for Biomedical Applications

**DOI:** 10.3390/md16080287

**Published:** 2018-08-18

**Authors:** Gratiela Teodora Tihan, Roxana Gabriela Zgarian, Elena Berteanu, Daniela Ionita, Georgeta Totea, Catalin Iordachel, Rodica Tatia, Mariana Prodana, Ioana Demetrescu

**Affiliations:** 1 Department of General Chemistry, Faculty of Applied Chemistry and Materials Science, University Politehnica of Bucharest, Polizu Street No.1, Bucharest 011061, Romania; gratielatihan@yahoo.com (G.T.T.); md_ionita@yahoo.com (D.I.); georgeta.totea@yahoo.com (G.T.); prodana_mariana@yahoo.com (M.P.); ioana_demetrescu@yahoo.com (I.D.); 2 Department of Biomaterials, National Institute of Research and Development for Biological Sciences, Bioproducts, Splaiul Independentei, No.296, Bucharest 060031, Romania; lili_berteanu@yahoo.com (E.B.); cataliniordachel@yahoo.com (C.I.); rodica.tatia@gmail.com (R.T.)

**Keywords:** chitosan, alkaline phosphatase, metal ion, hemolytic index, cell viability

## Abstract

In this paper, we present the fabrication and characterization of new chitosan-based membranes while using a new biotechnology for immobilizing alkaline phosphatase (ALP). This technology involved metal ions incorporation to develop new biopolymeric supports. The chemical structure and morphological characteristics of proposed membranes were evaluated by infrared spectroscopy (FT-IR) and the scanning electron microscopy technique (SEM). The inductively coupled plasma mass spectrometry (ICP-MS) evidenced the metal ion release in time. Moreover, the effect of Mg^2+^ on the enzymatic activity and the antibacterial investigations while using Gram-negative *Escherichia coli* and Gram-positive *Staphylococcus aureus* bacteria, hemolysis, and biocompatibility behavior were studied. Immobilizing ALP into the chitosan membranes composition followed by the incorporation of Mg^2+^ led to polymeric supports with enhanced cellular viability when comparing to chitosan-based membranes without Mg^2+^. The results obtained evidenced promising performance in biomedical applications for the new biopolymeric supports that are based on chitosan, ALP, and metal ions.

## 1. Introduction

In recent decades, the immobilization of active biomolecules on solid supports in order to increase the efficiency of separation and purification processes is an intensively investigated field of modern biotechnology [1,2,3,4]. Two major benefits of the immobilization process, such as easy separation of biomolecules from products and the possibility of biomolecular reuse can be identified. Over time, supports that are used to immobilize enzymes ranged from ceramics, metals, or metal oxides to chitin-like biopolymers [5], chitosan [6,7,8], agar, collagen [9], and gelatine [10,11]. Chitosan (CHI), which is derived from marine sources, is a polycationic polymer and a remarkable candidate for membrane production due to its reactivity, hydrophilicity, mechanical, and biocompatibility properties [12,13,14]. Structurally, CHI has a significant number of hydroxyl and amine reactive groups, which can lead to important functional properties in the development of biomaterials [15,16,17]. CHI-based materials are not toxic and have no allergenic effect in living body [18]. Also, these types of materials have shown antimicrobial activity against different groups of microorganisms, such as Gram-positive and Gram-negative bacteria [19,20,21,22]. Until now, it is not fully understood how the presence of chitosan provides the antibacterial effect, but numerous publications have demonstrated that the presence of amino groups from chitosan, and the molecular conformation, ensure the obtaining of a chitosan layer around the bacterial cell with an antibacterial effect [23]. In addition, recent important reviews have revealed the ability of CHI for drug encapsulation and controlled drug release [17], and the use of chitin and chitosan-based nanofibers in biomedical domains, such as bone tissue engineering, antimicrobial treatment, and medical implants [23].

In the bone tissue engineering field to induce bone regeneration, the combination of biomaterials with cells is very important. The biopolymeric support should be osteoconductive to ensure cell adhesion, migration, and proliferation [24]. The osteoinduction ability of CHI is increased by immobilizing enzymes [4,15,25]. Alkaline phosphatase (ALP) presence induced biomineralization, generating inorganic phosphate [26], and promoting cell growth and proliferation [25]. ALP, the enzyme of the hydrolases class (ALP, E.C. 3.1.3.1.), can be isolated from different animals and human beings tissue [27]. In humans, the highest concentrations of ALP are found in the liver, bones, kidney, and placenta. ALP is responsible for removing the phosphate group from several types of molecules, including nucleotides, proteins, and alkaloids. The enhancement of the enzyme properties and functional stability by immobilization techniques is important in various fields [25]. It is possible to improve the functional properties of the enzyme, such as stability, specificity, and activity, under different conditions, thereby increasing its applicability.

In 2009, Osathanon et al. described [26] a method to immobilize ALP on fibrin scaffolds controlling pore size and pore interconnection, with biological properties being demonstrated both in vitro and in vivo. They concluded that immobilized ALP fibrin scaffolds are good candidates for bone tissue regeneration. In 2015, thermosensitive injectable hydrogels that are based on CHI with sodium beta-glycerophosphate and Mg incorporated [8] were studied. Both hydrogels were loaded with ALP to induce hydrogel mineralization.

Our previous studies consisted in obtaining of different polymeric supports with good hemolytic activity and non-toxic effects, such as: collagen-based membranes with polyvinyl alcohol or polyethylene glycol [28,29,30] and also chitosan-based membranes with gelatine (GEL) [31] or collagen-based membranes with CHI and ALP [13]. The present study is a continuity of the biotechnology for protein immobilization in implant osteointegration, where we have obtained and characterized new CHI-based membranes with GEL and ALP, with and without metal ions as scaffolds for tissue engineering. To improve the bioperformance of the studied membranes, as novelty, the fabrication method of the CHI gel includes: a dialysis process to increase the pH value, and new ratios between CHI, GEL, and ALP, and different concentrations of metal ions. The structural characterization, enzymatic activity, and morphology of the new membranes were investigated and correlated with their antibacterial effect, hemocompatibility, and ability to support and promote cellular proliferation without changes of cells morphology.

## 2. Results

### 2.1. Fourier Transform Infrared (FT-IR) Spectroscopy

Infrared (IR) spectra of raw standard chitosan and ALP, and of chitosan-based membranes cross-linked and uncross-linked with glutaraldehyde GA, with or without ALP and metal ion were recorded. In order to evaluate the interaction between molecules, specific functional groups were identified.

**Figure 1:**
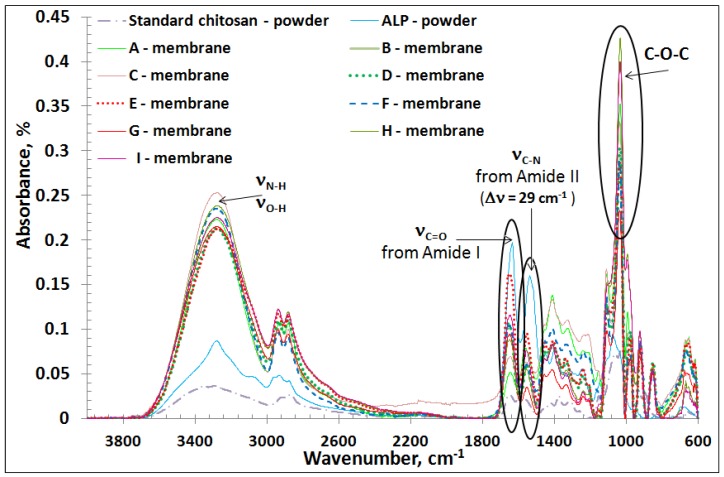
Infrared (IR) spectra of raw materials and studied membranes.

The IR spectrum of the standard CHI powder (Figure 1) presented absorption bands in the 3344 cm^−1^–3290 cm^−1^ domain, which were due to several vibrations, including the stretching vibration of hydrogen bonds (ν_O–H_) overlapped with the stretching vibration of N–H bond from the free amino group (–NH_2_) at C_2_ position of glucosamine. Other specific bands to CHI were recorded at 2915 cm^−1^ and 2873 cm^−1^, which were attributed to the stretching vibrations of C–H in –CH_2_ and in –CH_3_ groups, respectively. The absorption bands corresponding to the stretching vibration of C=O (ν_C=O_) from Amide I (O=C–NHR) and to the deformation vibration of NH_2_ (δ_N–H_) from Amide II appeared around 1654 cm^−1^, 1644 cm^−1^ and 1584 cm^−1^, confirming the partially deacetylated form of the standard CHI powder. The absorption band recorded around 1416 cm^−1^ was attributed to the stretching vibration of the C–N bond (ν_C–N_), and the band at 1375 cm^−1^ was assigned to the –C–O–H stretching of primary alcoholic group. The peak at 1315 cm^−1^ was attributed to the symmetrical deformation mode of CH_3_ [12]. Absorption band in the range 1150 cm^−1^–1060 cm^−1^ was assigned to the –C–O–C bond (glycosidic linkage). –CH_2_–OH was represented by a strong absorption band at 1025 cm^−1^, and the presence of the band at 895 cm^−1^ also demonstrates the polysaccharide structure.

When comparing the IR spectrum of standard CHI powder with that of membrane A based on CHI gel (Figure 1), the same characteristic bands, but at a higher intensity, were found.

The IR spectrum of GEL membrane (B) (Figure 1) evidenced the functional groups that are specific to amino acids [32]: the band at 3279 cm^−1^ assigned to the stretching vibration of N–H (ν_N–H_); the band at 2935 cm^−1^ attributed to the stretching vibration of C–H from –CH_2_ (ν_CHas_); the peak placed at 1646 cm^−1^ specific to Amide I, attributed to the stretching vibration of C=O (ν_C=O_) and coupled with COO^−^, the band corresponded to deformation vibration of N–H (δ_N-H_), and to the stretching vibration of C–N (ν_C–N_) revealed by the band at 1553 cm^−1^ from Amide II; the band at 1453 cm^−1^, corresponding to C–H bond deformation, and the band at 1239 cm^−1^ due to N–H bond deformation.

The IR spectrum of pure ALP (Figure 1) showed three characteristic bands, as follows: the absorption band at 1634 cm^−1^ corresponding to C=O (ν_C=O_) from Amide I, the bands at 1538 cm^−1^, and 1447 cm^−1^ corresponding to the deformation vibration of N–H (δ_N–H_) from Amide II.

The IR spectrum analysis of the CHI:GEL membrane cross-linked with GA (membrane C) (Figure 1) demonstrated the presence of absorption bands specific to the individual components, the formation of intramolecular and intermolecular hydrogen bonds and the electrostatic attraction between –COO^−^ of gelatine and –NH^3+^ from CHI. The shifting of Amide I and Amide II absorption band from standard CHI to smaller wavenumbers in the case of membrane C (1646 cm^−1^ and 1564 cm^−1^, respectively) also demonstrated the interaction with carboxyl groups of GEL [31,33]. Furthermore, the band corresponding to the C–O–C bond, which in standard CHI occurred at 1150 cm^−1^, in the IR spectrum of the membrane C, this band appeared at 1155 cm^−1^, but at a very low intensity, demonstrating cross-linking with GA [12].

To establish the influence of ALP on the membrane structure, the IR spectra of membranes C and F were compared with that of ALP (Figure 1). At approximately the same wavenumber, 3275 cm^−1^, the IR spectra of the two membranes C and F were similar, indicating the presence of hydrogen bonds and N–H amine groups in structure. The absorption bands registered in 2940 cm^−1^–2880 cm^−1^ domain were found at the same wavenumber (2937 cm^−1^ and 2882 cm^−1^) and with approximately the same intensity in both membranes C and F, being characteristic to the stretching vibrations of C–H (ν_C–H_) in the –CH_2_ and in –CH_3_ groups of the chitosan (ring). For both membranes C and F, the absorption bands corresponding to Amide I and Amide II were recorded, but at a much lower intensity than in the pure ALP case. Practically, the presence of ALP in the structure led to a decrease in the intensity of these bands, which confirmed the participation of functional groups of polymer in the reaction with GA and ALP.

By ALP inclusion, a significant shift of the Amide II band (1584 cm^−1^ in standard chitosan) to a smaller wavenumber (1555 cm^−1^ in membrane F) was registered. This behavior confirmed the interaction of CHI with GEL in the presence of the cross-linking agent, as well as the influence of the immobilized enzyme, which determined an important shifting (Δν = 29 cm^−1^).

To highlight the influence of GA on samples containing ALP, the IR spectra of the membranes D and F were studied (Figure 1). The presence of GA in membrane F led to an increase in the absorption band intensity at 3275 cm^−1^, indicating a better association by hydrogen bonding between the individual components.

In the IR spectra of the membranes D and E with different content of GEL and no cross-linking agent added, the high intensity of Amide I and also its shifting to a higher wavenumber than in the chitosan standard indicates a poor interaction between the functional groups of chitosan and gelatine with ALP.

Based on the spectral results registered for the membranes G, H and I, it was observed that the Amide II band moved from 1584 cm^−1^ (in standard CHI) to smaller wavenumbers ~1555 cm^−1^ (Figure 1). The addition of Mg^2+^ into the membranes composition evidenced a better interaction between components. Moreover, the influence of Mg^2+^ was demonstrated by other methods.

### 2.2. Enzymatic Activity for Samples with Different Metal Ion Concentrations 

The metal ions concentration presented in enzyme and membranes was highlighted by coupled plasma mass spectrometry (ICP-MS), and the influence of added metal ions on the enzymatic activity of ALP from bovine intestinal mucosa was studied. The results are summarized in Table 1.

It was found that before and after Mg^2+^ incorporation into the membranes, all of them contain metal ions, as Mg^2+^ and Zn^2+^ present in the ALP control sample. In addition, as the concentration of the added MgCl_2_ solution to the membranes composition increases, Mg^2+^ concentrations increases to higher values than 0.068 µg/mL, as it was in membrane F.

In order to evaluate the influence of metal ions on the enzymatic activity, ALP control as well as membranes with immobilized ALP with and without added metal ion were investigated (Table 1). A calibration curve was obtained by varying the concentration of p-nitrophenol, and the absorbance at a wavelength of 410 nm was read. One unit of enzymatic activity is able to catalyse the hydrolysis of 1 μmol of pNPP to pNP per min. When comparing the membrane F with membranes G, H and I, a different behavior was observed. The addition of various MgCl_2_ concentrations led to an increase of the phosphatase activity, the higher values being registered in the case of membranes H and I. The results are expected to have an important impact on the cell growth and proliferation.

### 2.3. Contact Angle Measurements 

The hydrophilic/hydrophobic capacity of a biomaterial has a significant role on controlling the cell growth and proliferation. It is well known that enhanced cell attachment is obtained when the surfaces are hydrophilic. The mean water contact angle values (CA) ± Standard Deviation for three replicates are presented. Therefore, in the case of membranes that are based just on CHI (membrane A) or GEL (membrane B), the CA values were 89.70 ± 1.11 and 60.10 ± 0.41, respectively, but in the case of membrane C, when both CHI and GEL were cross-linked with GA, the CA was 69.52 ± 0.38 indicating a moderately hydrophilic character of it. Immobilizing ALP into the membranes D, E, and F, CA values decreased to 19.51 ± 0.62, 21.53 ± 0.36 and 51.16 ± 0.38, respectively. The introduction of different concentrations of metal ion into the cross-linked membranes with immobilized ALP led to a decrease of CA values to 22.67 ± 0.45 (membrane G), 30.20 ± 0.22 (membrane H) and 34.58 ± 0.41 (membrane I), respectively, demonstrating pronounced hydrophilicity. It was concluded that hydrophobic character of the membrane was modified into moderate hydrophilic character or a pronounced one by ALP immobilizing and metal ion incorporation, suitable character for use these membranes as supports for cell growth and proliferation. Moreover, even the CA values that were recorded for membranes D, E, G, H, and I exhibited good hydrophilic character, required for good cell attachment, this may not be enough if the support is not well cross-linked, as is the case of the membranes D and E.

### 2.4. Scanning Electron Microscopy (SEM)

**Figure 2:**
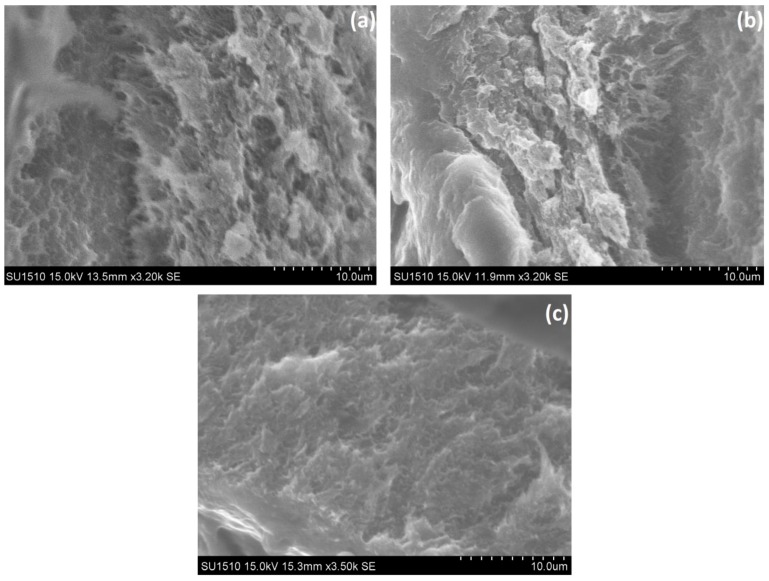
Scanning electron microscopy (SEM) images of membranes without alkaline phosphatase (ALP) and Mg^2+^: (**a**) chitosan (CHI); (**b**) gelatine (GEL); (**c**) CHI:GEL (1:1), glutaraldehyde (GA). Scale bar: 10 µm.

**Figure 3:**
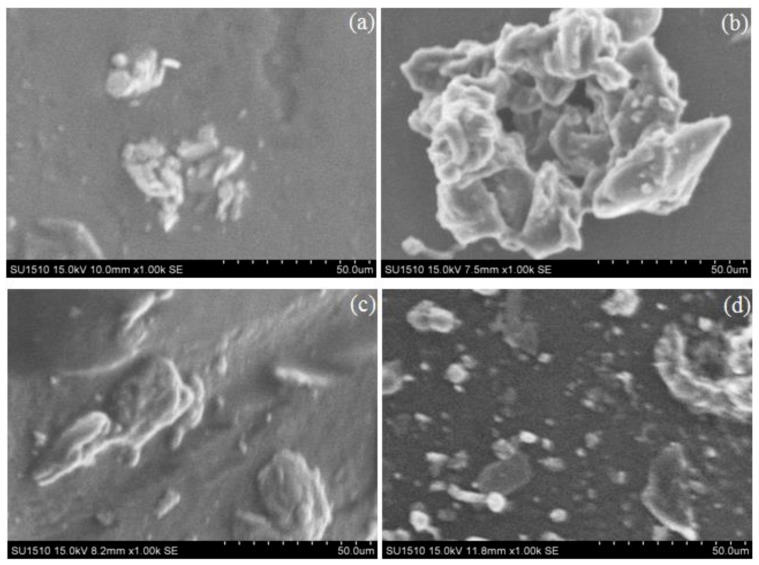
SEM images of membranes with ALP and Mg^2+^: (**a**) CHI:GEL (1:1), ALP, GA; (**b**) CHI:GEL (1:1), ALP, GA, 0.01% MgCl_2_; (**c**) CHI:GEL (1:1), ALP, GA, 0.1% MgCl_2_; and, (**d**) CHI:GEL (1:1), ALP, GA, 0.2% MgCl_2_. Scale bar: 50 µm.

Besides the chemical structure of polymeric supports, surface morphology was another important factor in achieving good cell attachment [34]. The effect of ALP and metal ions incorporation on chitosan-based membranes morphology was evaluated by SEM (Figure 2 and Figure 3).

For this purpose, Figure 2 shows the SEM images of the CHI-based membrane, GEL-based membrane and CHI:GEL:GA-based membrane before ALP and metal ions incorporation. In all these cases, rough surfaces were observed. After ALP and metal ions incorporation, Figure 3 revealed that crystal-like structures were formed on the surfaces. As shown in Figure 3a, few micro sized crystals with area between 4 µm and 56 µm were formed on a part of the membrane F surface. In the case of membranes G, H and I (Figure 3b–d) in which various concentrations of MgCl_2_ were added into the composition, micro sized crystals with different areas and distributions were observed. In the SEM image of the membrane G (Figure 3b) with 0.01% MgCl_2_, a large aggregate consisting in micro crystals with areas between 11 µm and 250 µm. Figure 3c evidenced the surface of membrane H containing 0.1% MgCl_2_, with few micro crystals with areas between 7 µm and 555 µm, which are distributed on a part of the surface. When 0.2% MgCl_2_ was added, as in the case of membrane I (Figure 3d), micro crystals with two types of sizes around 4 µm and 188 µm were distributed over the entire surface. As a result of combining CHI with ALP, CHI tends to form crystal-like structures. In addition, the incorporation of Mg^2+^ led to the formation of numerous micro sized crystals that were dispersed throughout the surface as the metal ion concentration increases. Several factors, like intra- and intermolecular hydrogen bonds, the electrostatic attraction, and the activation of the enzyme in the presence of Mg^2+^ contributed to this behavior. The way that the numerous micro sized crystals were distributed over the surface, especially in the case of the membrane I, can lead to good cell adhesion.

### 2.5. Antibacterial Activity

The antibacterial activity of CHI was widely studied, but the mechanism is not fully explained. Studies stated that the chelating activity of CHI has been involved as a possible mode of action or CHI might have intracellular targets, such as DNA [35], but also the CHI acted as a membrane perturbant [36]. The inhibition percent of bacteria growth on studied membranes is presented in Table 2 According to the literature [37], all of the studied membranes proved to be more effective for Gram-negative bacteria than Gram-positive bacteria. As expected, the most effective antibacterial activity was obtained in the case of membranes based just on CHI or GEL (membrane A, and membrane B, respectively). Referring only to the membranes F, G, H and I, which showed the best cross-linking between the components, a decrease of the antibacterial effect against both bacteria was observed. This behavior can be explained by the presence of immobilized ALP into the membranes composition. Moreover, the immobilization of ALP into the membranes, and also the incorporation of MgCl_2_ showed a slight increase in the antibacterial effect when an increased concentration of MgCl_2_ solution was added. The best antibacterial effect against *Escherichia coli (E. coli)* and also on *Staphylococcus aureus* (*S. aureus*) was recorded on membranes H and I, suggesting the positive influence of the Mg^2+^.

The antibacterial activity against *Escherichia coli* and *Staphylococcus aureus* showed a small antibacterial effect, but taking into account that all of these membranes do not contain antibiotics to ensure this effect, these results are important.

### 2.6. Hemolytic Study

The blood compatibility had a high relevance in all clinical procedures, the blood being the most important body fluid contacting wound dressing, cardiac valves, or implant. The interaction of blood with artificial materials was very complicated and not fully explained [38]. When blood was exposed to an artificial surface, the sanguine elements suffered major alterations, one of the most interesting being the destruction of red cells membrane, followed by cell lyses. Thus, the haemoglobin release was investigated to establish the hemocompatibility of the material.

Hemolysis tests were conducted in triplicate and the average hemolytic index (HI%) values ± Standard Deviation for three replicates were obtained, as follows: 2.9 ± 0.31 for membrane A, which fits into the slightly hemolytic category, and 0.22 ± 0.12 for membrane B which is non-hemolytic. Mixing CHI and GEL with GA (membrane C) or with ALP (membrane D), the hemolytic index significantly increased to 5.1 ± 0.21 (membrane C) and to 8 ± 0.52 (membrane D), respectively, indicating the hemolytic character of them. The hemolytic index decreased to 1.97 ± 0.08 (E) when more GEL was used, and to 2.19 ± 0.21 (membrane F) when CHI, GEL, and ALP were cross-linked with GA. Moreover, the addition of Mg^2+^ to membranes G, H, and I led to the obtaining of non-hemolytic supports, the hemolytic index being 1.6 ± 0.09 (membrane G), 1.5 ± 0.1 (membrane H), and 0.5 ± 0.17 (membrane I), respectively. Even if the low hemolytic index was obtained for membranes B, E, F, G, H and I, the membranes of interest remained G, H and I, which had demonstrated good cross-linking and good enzymatic activity as well as a non-hemolytic character.

### 2.7. In Vitro Biocompatibility

**Figure 4:**
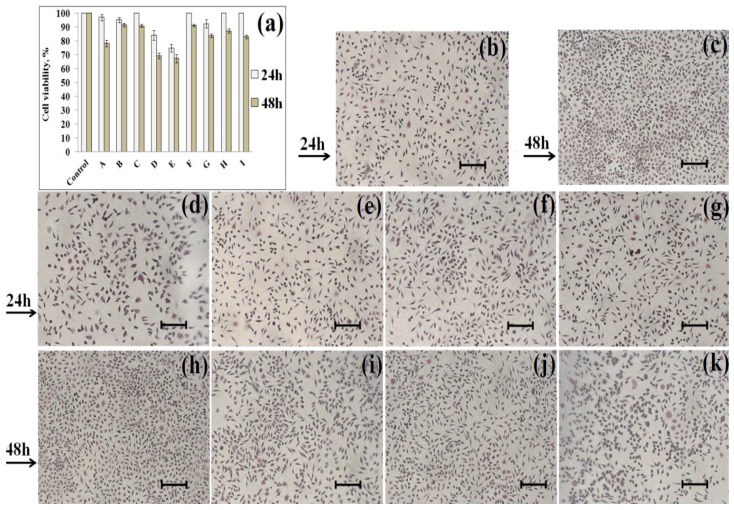
Cell viability and morphology at 24 h and 48 h: (**a**) Cell viability for Control and membranes at 24 h and 48 h; (**b**) cell morphology for Control at 24 h; (**c**) cell morphology for Control at 48 h; (**d**) cell morphology for CHI:GEL (1:1), ALP, GA at 24 h; (**e**) cell morphology for CHI:GEL (1:1), ALP, GA, 0.01% MgCl_2_ at 24 h; (**f**) cell morphology for CHI:GEL (1:1), ALP, GA, 0.1% MgCl_2_ at 24 h; (**g**) cell morphology for CHI:GEL (1:1), ALP, GA, 0.2% MgCl_2_ at 24 h; (**h**) cell morphology for CHI:GEL (1:1), ALP, GA at 48 h; (**i**) cell morphology for CHI:GEL (1:1), ALP, GA, 0.01% MgCl_2_ at 48 h; (**j**) cell morphology for CHI:GEL (1:1), ALP, GA, 0.1% MgCl_2_ at 48 h; and, (**k**) cell morphology for CHI:GEL (1:1), ALP, GA, 0.2% MgCl_2_ at 48 h. Scale bar: 50 µm.

The cell viability results from the MTT assay are presented in Figure 4. After 24 h of treatment, cell viability values were higher than 80% suggesting a good biocompatibility of the membranes, except for the membrane E, which proved to have a slightly cytotoxic effect (74.96%). When comparing the values that were recorded for membranes based on CHI and GEL with immobilized enzyme and metal ion (membranes G, H and I), which showed good cross-linking between the components, good hydrophilic character and good hemolytic index, the best percentages of viability were registered for membranes H and I (100%). It can be said that a large number of aggregates that were uniformly distributed on the entire surface of membranes H and I confirmed by SEM (Figure 3c,d) led to better cell viability than if the aggregates were formed only on certain areas.

After 48 h, membranes A, D and E manifested a slightly cytotoxic effect, inducing cell viability values between 50% and 80%, but all others membranes had non-cytotoxic effect. When comparing the results for membranes G, H and I, the highest viability at 48 h was obtained for membrane H (87.22%).

Cell morphology images obtained at 24 h and 48 h for culture treated with membranes F, G, H and I were represented in Figure 4. The morphology aspect of treated cells was similar with the normal morphology of the untreated NCTC mouse fibroblasts cells (Control). The cell density for culture treated with membrane F had a comparable level with untreated cells, while the level of cellular proliferation for the culture treated with membranes G, H and I was slightly lower, but without changes in morphology of the cells, indicating that all of these membranes were biocompatible with NCTC cells. Morphology images of the treated cells were suggestive results, thus confirming the MTT testing. As result of the biocompatibility tests it can be concluded that membranes H and I can be successfully used in the medical field, due to their ability to support and promote cellular proliferation.

## 3. Materials and Methods 

### 3.1. Materials

Commercial CHI powder (crab shell-derived) and bovine intestinal mucosa alkaline phosphatase (ALP) were purchased from Sigma-Aldrich (St. Louis, MO, USA). CHI powder, as prepared by the partial deacetylation of chitin in hot alkali, contains approximately 20% β1,4-linked *N*-acetyl-d-glucosamine (GlcNAc) and approximately 80% β1,4-linked d-glucosamine (GlcN), and has a viscosity >400 mPa.s (1% in acetic acid at 20 C). GEL powder from porcine dermis and tendons was purchased from Fluka BioChemika (Buchs, Switzerland). Glutaraldehyde (GA) 25% as cross-linking agent was from Merck (Kenilworth, NJ, USA). All of the reagents were of analytical grade.

### 3.2. Membranes Preparation

CHI powder was dissolved in acetic solution (acetic acid 2 M and sodium acetate 1 M) and let for stirring at 50 °C until the homogenous gel was obtained. The CHI gel in a concentration of 1% was then subjected to the dialysis process for 24 h. It was transferred to dialysis bags that were previously prepared according to the protocol: washing with tap water for 2–3 h, and with distilled water for 5 min at 65 °C, immersing in 0.2% sulfuric acid for 5 min, and finally washing with distilled water at 65 °C and cooling. The pH of the CHI after dialysis increased from 5.15 to 5.6. GEL powder was immersed in deionised water and heated at 60 °C. The obtained gelatine gel in a concentration of 1% had a pH as 5.56.

As reference, simple membranes like CHI membrane and GEL membrane were prepared (Table 3). The two individual components, CHI and GEL, were mixed in gravimetric ratios of 1:1 or 1:2 (g/g). Then, 5 mL ALP solution (1 mg/mL) was added under continuous stirring into the gel that was obtained by combining CHI with GEL. To a better inclusion of the enzyme in the substrate and its activation, a cross-linking process with 0.01% GA was used. After the immobilization of ALP, different concentrations of MgCl_2_ (0.01%, 0.1% and 0.2%) were added. The mixtures obtained were stirred, poured into Petri dishes, let for drying at 25 °C for 10 days, and finally, the membranes were obtained (Table 3).

### 3.3. FT-IR Spectroscopy

The Fourier transform infrared (FT-IR) spectra were achieved using a Perkin Elmer Spectrum 100 FT-IR spectrometer (Perkin–Elmer, Shelton, CT, USA) in the attenuated total reflection mode (ATR). ATR/FT-IR spectrum registered at room temperature between 4000 cm^−1^ and 600 cm^−1^ represents the average of 32 scans that were collected at a spectral resolution of 4 cm^−1^.

### 3.4. ICP-MS Measurement

The inductively coupled plasma mass spectrometry (ICP-MS) was performed on an ELAN DRC-e Perkin Elmer (Perkin–Elmer, Shelton, CT, USA). The samples were mineralized in concentrated HNO_3_ for 1 h and then diluted with distilled water. Calibration curves using multi-element standard containing 10 μg/mL (ppm) of Mg^2+^ and Zn^2+^ were used. The ALP solution was considered as reference sample.

### 3.5. Enzymatic Activity

Enzymatic activity was determined by the method of Ghiaci et al. [39]. Thus, 1 mL of the enzyme solution (1 mg/mL) was mixed with 1 mL of 2-amino-2-methyl-1-propanol (AMP) 0.1 M (ROTH), MgSO_4_ 2 mM, ZnSO_4_ 1 mM (pH 10) and 0.5 mL of pNPP (p-nitrophenylphosphate) 0.11 M (SIGMA-Aldrich, Dorset, UK) dissolved in AMP buffer. The ALP catalyses the hydrolysis of pNPP and produces an yellow compound as pNP (p-nitrophenol). All of the samples were incubated at 37 °C for 1 h, and the reaction was stopped by the addition of 2.5 mL of NaOH 0.5 M. The release of pNP was measured spectrophotometrically at 410 nm, while using an UV/VIS T80 equipment (PG Instruments Ltd, Lymington, UK). Allthe analyzes were performed in triplicate.

### 3.6. Contact Angle Measurements

The hydrophilic/hydrophobic balance of the membranes was evaluated at room temperature, by contact angle (CA) measurements with a Contact Angle Meter (Kyowa Surface Chemistry Co., Ltd, Tokyo, Japan), KSV instruments CAM 100 equipment (KSV Instruments, Helsinki, Finland). The volume of distilled water used was 10 μL and the CA value represents the average of three measurements ± Standard Deviation.

### 3.7. Scanning Electron Microscopy (SEM)

In order to characterize the morphological structure of the new membranes, SEM analysis was carried out while using SEM Hitachi SU1510 equipment (Hitachi High-Technologies Global, Tokyo, Japan). The membranes were deposited on the carbon band, and the visualization was made at 15.0 kV using the secondary electron detector (SE) (Secondary electron detector, Tokyo, Japan).

### 3.8. Antibacterial Activity

The antibacterial properties of materials against *Escherichia coli* (ATCC 25922) and *Staphylococcus aureus* (ATCC 25923) were evaluated by a turbidimetric method. The bacteria were cultured overnight on Columbia Agar +5% sheep blood at 37 °C, and the microbial suspensions (inoculums) were prepared in nutrient broth, adjusted to 0.5 McFarland units. The tested membranes were placed in polypropylene (PP) tubes and the inoculum was added. One PP tube was filled only with bacterial suspension and used as positive control. After mixing, the tubes were incubated at 37 °C for 24 h and then studied when visible signs of growth or turbidity appeared. The absorbance was read at 600 nm with an automated analyser CHEMWELL 6010(Awareness Technology, Inc., Palm City, FL, USA). The bacterial inhibition growth index (*I*%) was calculated whileusing the following formula:

(1) $$I(\%)=\frac{\left(C_{24}-C_{0}\right)-\left(S_{24}-S_{0}\right)}{\left(C_{24}-C_{0}\right)}\times 100$$

Where: “*C*” is the optical density at 600 nm (OD_600_) of inoculum read at time 0 (before incubation) and after 24 h; and, “*S*” is OD_600_ of inoculum in contact with the samples before and after incubation.

The experiment was carried out in triplicates for each tested membrane.

### 3.9. Hemolytic Study

In order to evaluate the interaction between human blood cells and various forms of chitosan based materials, hemcompatibility tests were performed according to ISO 10993-4 indications [40], while using the direct contact method as described in American Society for Testing of Materials ASTM F 756-00 [41]. All of the samples were incubated at 37 °C for 72 h in calcium and magnesium free phosphate buffered saline (CMF-PBS). The blood for determination of the hemolytic index was collected in sterile condition, from healthy volunteers group by venipuncture in Becton-Dickinson vacutainer containing sodium citrate (3.8%) in a ratio of 9:1. The group has volunteers with the same sex and approximately the same age and they signed an informed consent regarding the confidentiality data and the use of remaining biological fluid in scientific purpose. After incubation in CMF-PBS, the samples were placed in PP tubes and 0.5 mL of citrated blood was added. Positive and negative controls were prepared by mixing 0.5 mL whole blood with 3.5 mL PBS and 3.5 mL distilled water, respectively. All of the tubes were incubated for 3 h at 37 °C. For a better contact between blood and membranes, the samples were shaken by gentle inversion every 30 min. After incubation, the tubes were centrifuged at 2000 rpm for 15 min. The supernatant thus obtained was separated and introduced into the analyzer. Absorbance of negative control, positive control, and plasma obtained by contact with samples was read at 545 nm using a Chemwell 6010 spectrophotometer. The hemolytic index (*HI*%) was calculated while using equation 2.

(2) $$HI(\%)=\frac{\left({\mathrm{OD}}_{\mathrm{sample}}-{\mathrm{OD}}_{\mathrm{negative}\;\mathrm{control}}\right)}{\left({\mathrm{OD}}_{\mathrm{positive}\;\mathrm{control}}-{\mathrm{OD}}_{\mathrm{negative}\;\mathrm{control}}\right)}\times 100$$

Where: “OD_sample_” is the optical density of sample at 545 nm; “OD_negative control_” is the optical density of negative control at 545 nm; and, “OD_positive control_” is the optical density of positive control at 545 nm.

The experiment was achieved in triplicates for each tested membrane.

### 3.10. In Vitro Biocompatibility

Cell viability MTT (thiazolyl tetrazolium bromide) assay was used to highlight the viability of the culture that was treated with the tested sample. The MTT assay consisted in the reduction of mitochondrial dehydrogenase enzyme by tetrazolium bromide salt in metabolically active cells. In the reaction, purple-blue formazan crystals were obtained and dissolved in isopropanol for measuring the absorbance [42,43]. For viability assay and cell morphology examination a stabilized line of mouse fibroblasts NCTC (929 clones) cell was used. NCTC cells were cultivated at a density of 4 × 10^4^ cells/mL in Minimum Essential Medium (MEM) (from Sigma-Aldrich, (St. Louis, MO, USA), supplemented with 10% fetal bovine serum (from Biochrom) and 1% antibiotics (penicillin, streptomycin, and neomycin from Sigma-Aldrich). Hematoxyline, eosin, picric acid and formaldehyde used for cell morphology staining, and also thiazolyl tetrazolium bromide salt and isopropanol used for cell viability assay were purchased from Sigma-Aldrich.

To prepare the samples for biocompatibility assay, the membranes were cut to a size of 25 mm^2^ and sterilized by exposure to UV radiation for 8 h.

To induce adhesion, the cells were seeds in 24-wells plates and incubated in ABS 1500 biology security cabinet (Bioquell) in conditions of wet atmosphere, 5% CO_2_ and 37 °C for 24 h. After 24 h, the culture medium (0.5 mL per well) was replaced with fresh medium and the membranes were added (25 mm^2^ per well), with every sample being tested in triplicate. Three wells containing untreated cells were used as negative control culture and other three well containing culture medium only, were used to provide the blank control for absorbance reading. After 24 h and 48 h of incubation, cell viability assay by MTT was performed and the cell morphology of the treated cells was examined. For the viability assay, the culture medium in each well was replaced with MTT solution and the plates were incubated for 3 h at 37 °C. The MTT solution from the wells was replaced with isopropanol followed by gentle shaking to solubilise the formazan crystals. Absorbance of colored solution was read at 570 nm while using a microplate reader Berthold Mithras LB 940 (Berthold Technologies GmbH & Co KG, Bad Wildbad, Germany). The measured optical density is directly proportional to the number of viable cells present in the tested cell culture and the results were calculated, as follows:

(3) $$\mathrm{cell}\;\mathrm{viability}(\%)=\frac{{\mathrm{OD}}_{\mathrm{sample}}}{{\mathrm{OD}}_{\mathrm{control}}}\times 100$$

where: “OD_sample_” is the optical density of sample at 570 nm; and, “OD_control_” is the optical density of control at 570 nm.

Untreated cells served as control considered as 100% viable cells.

Cell morphology examination of treated culture was performed by replacing the medium and samples from the wells with Bouin solution (7.5 mL picric acid moistened with water, ≥98% and 2.5 mL formaldehyde 40%) for the fixation of the cells, followed by staining with Hematoxilin–Eosin. Using an optical microscope Carl Zeiss Axio Observer D1 (Carl Ziess, Oberkochen, Germany), the cell morphology was visualized and the images were taken with a digital camera Axio Cam MRc (Zeiss, Germany).

## 4. Conclusions

In the present study, using biological components, changing the chemical structure and the surface morphology of the biopolymeric supports, the antibacterial activity and biocompatibility were improved. The inclusion method for the immobilization of ALP on CHI-based membranes and the incorporation of Mg^2+^ were done in order to combine their advantages in increasing the applicability in medical domain. Biopolymeric supports with a pronounced hydrophilic character and increased enzymatic activity were prepared. The presence of ALP and Mg^2+^ induced an enhanced cellular viability, which is beneficial for the management of bone tissue regeneration. By correlating the data obtained, the best cell vitableability at 24 h and 48 h, without changes in morphology of the cells, was recorded for membranes H and I, which also showed an antibacterial effect and non-hemolytic character, required characteristics for their use in medical applications.

## Figures and Tables

**Table 1:** Enzymatic activity and metal ion concentrations.

Sample	Metal Ion (µg/mL)		Enzymatic Activity (U/mg/min)
	Mg^2+^	Zn^2+^	
ALP (powder)	0.151	0.107	474
F ^*^ CHI:GEL (1:1), ALP, GA	0.068	0.038	91
G ^*^ CHI:GEL (1:1), ALP, GA, 0.01% MgCl_2_	0.091	0.049	122
H ^*^ CHI:GEL (1:1), ALP, GA, 0.1% MgCl_2_	3.750	0.057	286
I ^*^ CHI:GEL (1:1), ALP, GA, 0.2% MgCl_2_	5.812	0.081	237

F ^*^—Cross-linked chitosan membrane with immobilized ALP; G ^*^—Cross-linked chitosan membrane with immobilized ALP and 0.01% MgCl_2_; H ^*^—Cross-linked chitosan membrane with immobilized ALP and 0.1% MgCl_2_; I ^*^—Cross-linked chitosan membrane with immobilized ALP and 0.2% MgCl_2_.

**Table 2:** The bacterial inhibition growth index of studied membranes.

Bacteria	Inhibition of Bacteria Growth on Membranes (%)						
	A	B	C	F	G	H	I
*E. coli*	44.82 ± 1.68	36.01 ± 1.51	36.78 ± 1.45	11.87 ± 1.43	12.49 ± 2.06	14.55 ± 1.72	16.72 ± 1.12
*S. aureus*	38.98 ± 1.52	28.61 ± 1.28	32.61 ± 1.48	9.52 ± 2.10	10.96 ± 1.82	12.42 ± 2.08	12.05 ± 1.86

All values are expressed as mean ± Standard Deviation for three replicates.

**Table 3:** Membranes composition.

Membrane	Composition
A	CHI
B	GEL
C	CHI:GEL (1:1), GA
D	CHI:GEL (1:1), ALP
E	CHI:GEL (1:2), ALP
F	CHI:GEL (1:1), ALP, GA
G	CHI:GEL (1:1), ALP, GA, 0.01% MgCl_2_
H	CHI:GEL (1:1), ALP, GA, 0.1% MgCl_2_
I	CHI:GEL (1:1), ALP, GA, 0.2% MgCl_2_
